# Radiofrequency Coblation-Assisted Transoral Surgery for the Treatment of Oropharyngeal Squamous Cell Carcinoma: A Comparative Study with Open Surgery

**DOI:** 10.1155/2023/7487306

**Published:** 2023-02-08

**Authors:** Xiaowan Du, Xin Zhao, Junbo Zhang, Chi Zhang, Shuifang Xiao, Tiancheng Li

**Affiliations:** ^1^Department of Otolaryngology, Head and Neck Surgery, Peking University First Hospital, Beijing, China; ^2^Department of Otolaryngology, Head and Neck Surgery, Qingdao University Medical College Affiliated Yantai Yuhuangding Hospital, Qingdao, Shandong, China

## Abstract

**Objective:**

Radiofrequency coblation (RFC) is a relatively new method that has opened up new perspectives in treating oropharyngeal squamous cell carcinoma (OPSCC). Our study was designed to explore the feasibility and effectiveness of RFC-assisted transoral surgery (RFC-TOS) for primary OPSCC.

**Methods:**

Sixty-nine cases of OPSCC from February 2005 to November 2020 were retrospectively analyzed, including 31 in the RFC-TOS group and 38 in the open surgery group. No difference was observed in demographic and oncological characteristics.

**Results:**

The significance between the RFC-TOS group and the open surgery group was proved in intraoperative bleeding volume (34.10 ± 10.10 ml vs. 193.68 ± 21.00 ml, *P* < 0.001), durations of surgery (79.58 ± 8.45 min vs. 217.87 ± 17.65 min, *P* < 0.001), time to resume oral feeding (1.64 ± 0.41 d vs. 11.58 ± 1.41 d, *P* < 0.001), duration of hospitalization (7.84 ± 0.66 d vs. 15.66 ± 1.62 d, *P* < 0.001), and the total costs (22846.22 ± 1821.55¥ vs. 41792.24 ± 4150.86¥, *P* < 0.001). The rates of 5-year overall survival (OS), 5-yeardisease-specific survival (DSS), and 5-year local control rate (LC) were 69.1%, 71.7%, and 75.7%, respectively, in the RFC-TOS group and 71.0%, 73.4%, and 73.7% in the open surgery group (*P* > 0.05).

**Conclusions:**

RFC-TOS is a feasible alternative transoral approach for OPSCC. The reported perioperative and oncologic outcomes are satisfactory.

## 1. Introduction

Oropharyngeal squamous cell carcinoma (OPSCC) accounts for 90% of oropharyngeal malignancies [1], and the number of OPSCC patients is still increasing [2]. According to the National Comprehensive Cancer Network (NCCN) guidelines, the therapeutic options for OPSCC are surgery with or without adjuvant therapy and radiotherapy with or without chemotherapy. The adverse effects of nonsurgical treatments and recent technical innovations have prompted a new trend in surgical approaches [3]. Surgical treatment approaches have changed dramatically with a trend of minimally invasive surgery, especially for early-stage OPSCC. Traditional open surgery for such malignancies is associated with high complication rates, affecting speech and swallowing and changing appearance [4, 5]. During the past decades, transoral surgeries, including transoral laser microsurgery (TLM) and transoral robotic surgery (TORS), have altered the surgical landscape for resecting oropharyngeal malignancies. Such procedures are characterized by less trauma, excellent function preservation, fewer complications, and improved quality of life [3–6].

Laser, though widely used in TLM for the treatment of selectable tumors located in multiple regions of the upper aerodigestive tract [6–10], has its own limitations, including low efficiency in hemostasis and tissue manipulation, resulting in surgical difficulties and prolonged operation time [11–13]. These limitations are more obviously observed when treating oropharyngeal tumors, as these tumors are always associated with large volumes and abundant blood supplies [5]. The other limitations included the 2-dimensional cutting plane, serious thermal damage, and the risk of a catastrophic laser fire [12, 13]. TORS is another minimally invasive procedure that was approved by the FDA for treating early-stage OPSCC in 2009, with excellent visualization, decreased line of sight issues (using a 30° endoscope), improved range of motion (360° robotic arm movement), precise cutting, and easier en bloc resection [14–20]. Nevertheless, it lacks haptic feedback, and it is hard to popularize due to its extremely high cost of purchase and maintenance.

Radiofrequency coblation (RFC) is a relatively new method that has been widely used in several pharyngeal surgical procedures [18], including tonsillectomy, uvulopalatopharyngoplasty, and some tongue or tongue base procedures [21–24]. Its advantages include high cutting and hemostatic efficiency, less thermal damage, good haptic feedback, relief of postoperative pain, and lack of charring, making it an attractive choice in transoral surgery for treating OPSCC.

Currently, only very small clinical study data supported the feasibility of RFC-assisted transoral surgery (RFC-TOS) in treating oropharyngeal cancer [12, 25]. This retrospective study was conducted to further explore the safety, feasibility, and effectiveness of RFC-TOS in treating OPSCC compared with open surgery.

## 2. Materials and Methods

### 2.1. Patients

Between January 2005 and December 2020, a total of 31 patients with *T*1–3 stage OPSCC who successfully underwent RFC-TOS with or without postoperative adjunct radiotherapies at Peking University First Hospital were included for analysis (RFC-TOS group). Meanwhile, an additional 38 patients with *T*1–3 stage OPSCC who underwent open surgery with or without postoperative adjunct radiotherapies during the same period at Peking University School of Stomatology were included in the control group (open surgery group). The inclusion criteria were patients aged ≥18 years with no previous treatment history. The exclusion criteria included patients with a prior history of head and neck aerodigestive tract malignancy, distant metastasis, or multiple primary tumors outside the oropharynx during the presentation. The baseline, perioperative, and prognostic data of these two groups of patients were collected for analysis. The study has been approved by the Institutional Ethics Committee of Peking University First Hospital (approval number 2019-264) and was carried out following the Declaration of Helsinki.

### 2.2. Surgical Procedure

Open surgery procedures included transcervical, trans-hyoid approaches, or even partial mandible resection. In the RFC-TOS procedure, patients underwent surgery by transnasal endotracheal intubation under general anesthesia. Boyle-Davis mouth gag, molar mouth gag, or FK retractor were used to expose the surgical fields. EIC8870-01 coblation wand (Arthrocare Corp.; Austin, Texas, USA) or MC401 coblation wand (MECHAN; Chengdu, Sichuan Province, China) were used for tumor resection. The console was set for 7-8 (coblation) or 5 (coagulation). Primary tumors were removed by transoral “en bloc” resection. Frozen sections were applied to ensure surgical margins of at least 5 mm and, if possible, 10 mm for all intraoperative tumor resections. In certain cases with large tumors, it is not possible to ensure 5–10 mm margins; therefore, *R*0 resection status should be ensured. To prevent persistent postoperative nasopharyngeal reflux, patients with large oropharyngeal defects underwent reconstructive procedures using a buccal mucosal flap or hard palate flap. All patients staged cN + underwent concurrent selective neck dissections.

### 2.3. Adjunct Therapies and Follow-Up

Postoperative radiotherapy was suggested when the incisional margin was reported to be less than 5 mm from the tumor edge or when extranodal extension (ENE) was positive. There were 13 patients in the RFC-TOS group and 15 patients in the open surgery group who received postoperative radiotherapy with a dose of 50–60 Gy. All patients were regularly followed up in the outpatient department with physical examinations, neck ultrasounds, endoscopes, and enhanced CT. Meanwhile, the patients were also followed up by telephone regularly regarding the postoperative quality of life and potential complications that affect their speech and swallowing.

### 2.4. Statistical Analysis

Pearson's chi-square test or Fischer's exact test was applied to compare categorical variables. The *t*-test or nonparametric test was used for continuous variables. The Kaplan–Meier method was employed to analyze the survival outcomes, and the comparison was assessed by a log-rank test. Statistics significance exists when *P* < 0.05. Statistical analyses were performed using MedCalc software (MedCalc version 18.11.3; MedCalc Software, Ostend, Belgium).

## 3. Results

The comparisons of baseline characteristics between RFC-TOS and open surgery group patients are shown in Table 1. No significant differences were found in gender, age, tumor sites, tumor stages, pathological differentiation, p16 positive rate, and postoperative adjuvant radiotherapy (all *P* > 0.05).

Perioperative data were analyzed and compared between these two groups, as shown in Table 2. The mean intraoperative bleeding volume, operation time, recovery of oral feeding time, hospital stay days, and total costs in the RFC-TOS group were significantly lower than those in the open surgery group (all *P* < 0.05).

**Figure 1:**
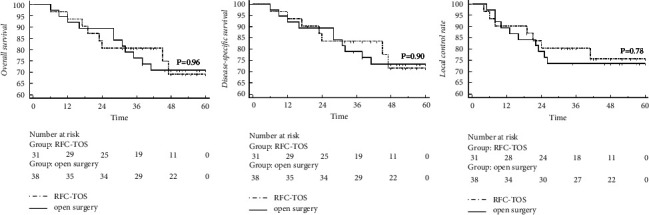
The KM curves of OS, DSS, and LC and the oncologic comparison between the RFC-TOS and open surgery groups.

Only one patient in the RFC-TOS group dropped out in the sixth month. The median follow-up periods were 42 months (6–151 months) in the RFC-TOS group and 50.5 months (6–142 months) in the open surgery group. As shown in Figure 1, no significant differences were found between the RFC-TOS and open surgery groups (all *P* > 0.05) regarding 5-year overall survival (OS) (69.1% vs. 71.0%), 5-yeardisease-specific survival (DSS) (71.7% vs. 73.4%), and 5-year local control rate (LC) (75.7% vs. 73.7%).

Postoperative complications in the open surgery group included 1 case of asphyxia (2.6%) because of a swollen tongue base (emergency tracheotomy needed). No short-term complications were found in the RFC-TOS group. At the end of the follow-up period, there were 3 cases of mild nasopharyngeal reflux (7.9%) and 1 case of taste loss (2.6%) in the open surgery group, while in the RFC-TOS group, 2 cases (6.5%) of mild nasopharyngeal reflux occurred occasionally and 1 case of taste loss (3.2%) was recorded. No long-term complications that affected speech, swallowing, or breathing were reported in both groups.

## 4. Discussion

RFC is considered a relatively new technique applied in transoral surgeries. Since its first technical descriptions and feasibility reports, only a few studies have reported perioperative outcomes for OPSCC. Among them, Carney et al. and Hofauer et al. included 10 and 12 cases of oropharyngeal malignancy resection, respectively [12, 25]. Neither of them reported long-term oncological results. Our study, while retrospective, is the first one focusing on OPSCC with a substantial population (*N* = 69), including 31 in the RFC-TOS group, and a relatively long follow-up.

The present study confirmed that, compared to open surgery, RFC-TOS evidently had favorable outcomes in terms of less intraoperative blood loss, shorter surgical time, rapid recovery of oral feeding, shorter length of hospital stays, and an economically friendly cost. Oncologic outcomes were also proven to be comparable to open surgery. These results suggest the feasibility and effectiveness of RFC-TOS in treating OPSCC.

Similar surgical data of TLM and TORS for *T*1 to *T*3 stage OPSCC resection were seldom published previously. Sievert et al. reported the blood losses of TLM and TORS as 121.5 ± 109.3 ml and 102.2 ± 76.9 ml, respectively [13]. Hofauer et al. reported a mean value of bleeding of 2.31 (SD = 0.82) for RFC-TOS treating oral and oropharyngeal tumors (*n* = 25) and described it as self-limiting in most of the cases [25]. In this study, the blood loss was 34.10 ± 10.10 ml (*n* = 31). Although the precise values of the blood loss and operation time are incomparable, they might suggest the correlations with hemostasis efficiency in different cutting instruments. The sealing capacities of the CO_2_ laser, monopolar scalpel, and RFC were reported, separately, as 1-2 mm, 2 mm, and 7 mm, measured by vessel diameters [3]. Because of the abundant blood supply in the oropharynx, it might be possible that RFC-TOS is more favorable for OPSCC, partially due to its better hemostasis capacity. In addition, operation times of TLM and TORS were reported as 140 ± 59 min (*n* = 10) and 186 ± 54 min (*n* = 9)^13^, while in this study, it was 79.58 ± 8.45 min in RFC-TOS (*n* = 31). Carney et al. suggested that RFC allowed for a much faster resection time than the CO_2_ laser for the resection of head and neck malignancies (*P*=0.017), especially in the oropharynx (*P*=0.007) [12]. RFC-TOS also showed sufficient competence in tissue manipulation without high thermal damage [11]. This suggested RFC-TOS is an attractive alternative technique in transoral surgery for oropharyngeal lesions [3, 12].

Cost is one of the important factors that determine whether a treatment can be widely accepted by patients, which is mostly influenced by equipment [26]. Analyses of costs were rare. Sievert et al. reported incurred costs of a significantly higher average amount of 5719.20 ± 3611.79 euros for TORS and 2856.35 ± 1439.75 euros for TLM (*P*=0.002) treating OPSCC [13]. Dombrée et al. analyzed the total costs for supraglottic open, TLM, and TORS approaches and confirmed them to be 3,349 euros (3,193–3,499 euros), 3,461 euros (3,207–3,664 euros), and 5,650 euros (4,297–5,974 euros), respectively [26]. These may suggest that the order of costs from highest to lowest would be TORS, TLM, and open surgery. Our study showed the total costs for open surgery and RFC-TOS are 22846.22 ± 1821.55¥ and 41792.24 ± 4150.86¥ (*P* < 0.001), respectively. It might indirectly indicate that RFC could be the most economical and practical method of all, which would greatly help the popularization of this method.

Less precise cutting is one major disadvantage of RFC. Carney et al. first proposed that the RFC probe design limits evaluating the surgical margin [12]. Hofauer et al. reported an average width of 1593.75 *μ*m of the coagulation zones of RFC in resecting oral and oropharyngeal lesions [25]. Wider margins are needed in OPSCC, where the most frequent identification of a clear margin was >5 mm on microscopic examination [18, 27]. The definition is also included in the National Comprehensive Cancer Network (NCCN) guidelines for head and neck cancer. Therefore, the disadvantage of RFC is acceptable for OPSCC resection.

In general, the oncological results of RFC-TOS treating *T*1 to *T*3 stage OPSCC (5-OS 69.1%, 5-DSS 71.7%, and 5-LC 75.7%) are in line with the prognosis of current publications of other transoral surgeries [13, 28–30]. Besides, the oncological results showed no significant difference compared with open surgery, namely the standard procedure.

However, RFC-TOS for OPSCC is considered a challenging and skilled procedure suitable for selected OPSCC patients. Rigorous indication, adaptation, and regulation of operation are strongly recommended.

The limitations of our investigation need to be addressed. The study was designed to evaluate the feasibility and oncological prognosis of RFC-TOS for OPSCC as a retrospective study and therefore was not planned as a blinded and controlled clinical trial, which might decrease the power of this investigation. In addition, data were collected from two different medical centers, which could have an impact on the comparison of perioperative and oncological results. Another limitation is that the comparison of RFC-TOS directly with TLM or TORS, instead of open surgery, could be more illustrative, which is the direction of our future work.

## 5. Conclusions

RFC could be an alternative tool for resecting OPSCC. Compared with traditional open surgery, RFC-TOS could offer comparable oncologic outcomes. OPSCC patients who underwent such surgery also showed rapid recovery, a low complication rate, and excellent functional outcomes. The superiority in economical cost and operation difficulty also makes RFC-TOS an attractive choice for treating OPSCC.

## Figures and Tables

**Table 1:** The baseline patient characteristics and comparison between the RFC-TOS and open surgery groups.

	RFC-TOS (*n* = 31)	Open surgery (*n* = 38)	*P* value
Gender			
Male	24 (77.4%)	29 (76.3%)	0.91
Female	7 (22.6%)	9 (23.7%)	
Age	56.3 ± 7.9	58.2 ± 10.6	0.41
Tumor site			
Tonsil/lateral wall	20 (64.5%)	22 (57.9%)	0.74
Soft palate	5 (16.1%)	9 (23.7%)	
Base of tongue	6 (19.4%)	7 (18.4%)	
*T* stage			
*T*1	8 (25.8%)	8 (21.1%)	0.63
*T*2	21 (67.7%)	25 (65.8%)	
*T*3	2 (6.5%)	5 (13.2%)	
N stage			
*N*0	22 (70.9%)	28 (73.7%)	0.80
*N*+	3 (9.7%)	10 (26.3%)	
Clinical stage			
I-II stage	20 (64.5%)	23 (60.5%)	0.74
III-IV stage	11 (35.5%)	15 (39.5%)	
Differentiation			
High	15 (48.4%)	15 (39.5%)	0.76
Moderate	12 (38.7%)	17 (44.7%)	
Poor	4 (12.9%)	6 (15.8%)	
p16 result			
p16+	18 (58.1%)	21 (55.3%)	0.82
p16−	13 (41.9%)	17 (44.7%)	
Radiotherapy			
Yes	13 (41.9%)	15 (39.5%)	0.84
No	18 (58.1%)	23 (60.5%)	

**Table 2:** Perioperative results and comparison between the RFC-TOS and open surgery groups.

	RFC-TOS (*n* = 31)	Open surgery (*n* = 38)	*P* value
Bleeding volume (ml)	34.10 ± 10.10	193.68 ± 21.00	<0.001
Duration of surgery (min)	79.58 ± 8.45	217.87 ± 17.65	<0.001
Time to resume oral feeding (d)	1.64 ± 0.41	11.58 ± 1.41	<0.001
Duration of hospitalization (d)	7.84 ± 0.66	15.66 ± 1.62	<0.001
Total hospital cost (¥)	22846.22 ± 1821.55	41792.24 ± 4150.86	<0.001

## Data Availability

The data supporting the findings of this data are available from the corresponding authors upon reasonable request.
